# A short cultural history of the UK Renal Registry 1995–2020

**DOI:** 10.1186/s12882-020-01997-1

**Published:** 2020-08-12

**Authors:** Eric John Will

**Affiliations:** Ex-Leeds Teaching Hospitals NHS Trust, England, UK

**Keywords:** Renal Registry, NHS, Clinical informatics, Guidelines, Quality improvement

## Abstract

The Renal Association UK Renal Registry (UKRR), established in 1995, has reflected the development of Nephrology within the NHS over 25 years. It has been gradually enlarged to provide a formal agency for a range of consensus initiatives. It remains the source of the national epidemiology of renal replacement, feeding NHS infrastructures and Health Services Research. An extension into acute and chronic kidney disorders is in hand. As a template for medical audit it has contributed to a quality improvement ethos derived from several methodologies. It now offers a multifaceted virtual platform for special interest groups and patient-centricity. Its transformation demonstrates one of the compromises that have permitted specialty development within the inconstant envelope of the NHS.

If not always a bellwether, the clarity, form and scale of kidney disease provision still qualifies the UKRR as a demonstrator of healthcare possibilities to Medicine, Clinical Informatics and the NHS.

## Background

There are several histories that could be presented for the 25th anniversary of the adult elements of the UK Renal Registry (UKRR). The traditional account of organisation, finance, personnel and products by the Renal Association (RA) would be complemented by a discussion of the evolution of statistics and presentation, say, or an attempt to discriminate an overall contribution to patient care [1, 2]. A less daunting task is the consideration here of the cultural milieu through which it has travelled. The survival of the Registry in the frequently reorganised UK National Heath Service (NHS) is probably down to its roots in the RA, together with the flexibility of the registry construct as an agency, a kind of meme. This cultural history is offered as a means of examining those likelihoods. While always denoted a ‘driver’ of change, the UKRR has itself been ‘driven’ by various cultural contingencies.

## Main text

To begin, Renal Medicine is a readily definable medical subspecialty. Diagnosis is based on laboratory features and treatment effected by the well-characterised processes of dialysis and transplantation. The professional roles, of setting (with the patient) the healthcare task and adjusting treatment, are clear cut, especially after the reformulation from the US of acute and chronic renal failure, as acute kidney injury (AKI) and chronic kidney disease (CKD) respectively, in the early millennium [3, 4]. The NHS managerial obligations, to enable clinical effort and sustain treatment processes, are also well-defined, as inferred by the wholesale commercialisation of the haemodialysis sector in the USA. This coherence, and a predominant numerical basis, gave the clinical specialty a head start in digital development and has permitted subsequent, quite independent, innovation [5].

It might be expected that this neat specialty profile would reflect the zeitgeists of Medicine and the NHS. The gradual transition of the NHS, from a government enabled, professionally determined entity after 1947 to one increasingly managed and planned, has indeed been manifest in the cultural history of the registry and the constituencies that it has served.

The European Dialysis and Transplant Association Registry (EDTA-R) had rehearsed the role of a mirror to the development of renal replacement treatments (RRT) after 1965. Their report on renal unit data returns had pride of place as the introduction to each annual European meeting. The substitution of electronic for paper data returns to EDTA-R was demonstrated from two dozen UK renal units in the late 1980s. It was that clinical IT capability that made an entirely electronic national renal registry feasible in 1995 [5]. The particular stimulus for the registry initiative in England, by a reluctantly political Renal Association, had been the need for continuous expression of the ever-expanding demand for dialysis to the Department of Health. In the event, the registry-presented core epidemiology of renal replacement was augmented in the database by an equal component of comparative medical audit of the readily available laboratory variables (a similar, more limited, template having been piloted in Scotland) [6]. Specifically, US authors had come to express dialysis ‘dose’ conveniently through blood urea concentration (1985) and relate survival to Serum Albumin levels (1990), both of which associated laboratory variables directly with clinical outcome [7, 8]. Nephrologists were also in the process of learning how to use Erythropoietin to correct renal anaemia. A complementary document of minimum ‘Standards and Audit Measures’ was produced by the RA/RCP, later renamed ‘Guidelines’. Their satisfactory achievement was left then, and subsequently, to local efforts, reflecting the medical culture of the time. The ultimate rationale of the registry was presented conventionally as a resource contributing to patient care. The registry repository of clinical activity and laboratory data painted national pictures of renal replacement in annual reports from 1998 onwards [2].

In the background was the intention within the NHS, after the Griffiths Report of 1983 and *Working for Patients* (1989), to acquire healthcare information to permit the comprehensive management of the service, not least the split between healthcare ‘Purchasers and Providers’. [9, 10] Both clinicians and the NHS responded to societal concerns for ‘getting it right’ and demonstrating that. The UKRR provided then a windfall of data, piloting that slowly developing objective in a common (RA and NHS) cause. The joint benefit was acknowledged financially by the institution of an annual patient capitation fee resourced from NHS renal unit budgets (1998), after the initial public/company-donated sponsorship of the foundation. The settled title of ‘Registry’ rather than ‘Register’ was arbitrary but prescient (vide infra) and from the first was created as a quietly hybrid professional/NHS collaboration.

While the EBM movement of the early 1990s concentrated on improving the medical skillset, in the light of Bayesian statistics for example, it was fostered by the examination of practice through audit (effectively, monitoring various elements of Donbedian’s structure, process and outcome triad). The NHS investment after 1989 in decentralised, piecemeal, medical (then broader ‘clinical’) audit was rationalised by the redirection of audit finance to ‘Purchasers’ in 1994. Quality Assurance was the prominent concern of the time but it was the US-derived Continuous Quality Improvement (CQI) philosophy/ideology, that had swept through US nephrology from the mid-1990s, which attracted NHS attention towards the turn of the century. The direction of development was reinforced by the reporting of aggregate patient hazard in the US healthcare system and the introduction of ‘Clinical Governance’ to the NHS [11–13]. CQI held an explicit promise of mitigating any persisting tension between clinicians and NHS management, by offering a shared mission of continuous change in healthcare. The suggestion of the NHS as an ideal demonstrator for CQI was plausible, although it was bound to insert workforce aspirations into a corporate command and control ethos. Usefully, CQI could also be said to complement EBM, as a uniform and comprehensive effector arm. A semantic and practical dovetailing is still ‘ongoing’ [14, 15]. For example, the NHS Healthcare QI Partnership (2008-)(currently HQIP, as part of the National Clinical Audit and Patient Outcomes Programme – NCAPOP) is largely concerned with national specialty audit exercises, arguably a residue of EBM. Rather inevitably, the UKRR as databank and agency came to straddle both approaches.

The National Renal Reviews of the early 1990s presaged the NHS Renal National Service Framework (R-NSF) of the early millennium, designed with healthcare commissioning in mind. It offered an attractive way forward for the specialty, where several longstanding issues offered an immediate substrate for examination (e.g. patient transport, patient choice of RRT). It started a national effort to bring all units toward the practice of the best. The comprehensive, multidisciplinary, scope of the R-NSF (2004–5) mobilised and powerfully reoriented specialty self-consciousness in and through the various contributors to the ‘Kidney Alliance’ (2001). The appointment of a central authority in the form of a co-opted renal ‘Tsar’ (2007) was received by the specialty more in terms of useful enablement than coercion. The focus of both the R-NSF and the UKRR remained on standards and comparative audit and it was only in 2006 that QI was mentioned in the annual registry report (Report 9, Ch 2, Introduction) [2]. Latterly the R-NSF promoted ‘Quality Requirements’ and introduced ‘action learning sets’, but it was Audit-feedback rather than CQI philosophy and methods that remained the predominant registry and medical ethos.

The UKRR reports of the time documented a wide variation of unit results and outcomes, the origins of which were incompletely understood [16]. Sophisticated medical QI efforts offered from the UKRR, based on Statistical Process Control, did not gain traction in the nephrological constituency of the time (2007 Report 9, Ch 2) [2]. It seems fair to say that, outside of standard procedures, there was limited interest in cohort-wide policies of clinical management in the multi-consultant units, as possibly compromising the focus on individual patient care. However, there was a noticeable incapacity in the translation of some areas of formal advice into practice, the very arena of CQI. The contemporary literature of methodologies to design compliant unit-wide results is sparse and exposes the demanding complexity of any successful attempts [17–19]. The point prevalent, aggregated laboratory results were data distributions that corresponded imperfectly to the dynamics of cyclical variables and the pitch of guideline assertions (which generally did not advise any means of achievement). Except for dialysis dose (Urea Reduction Ratio, URR) and Haemoglobin (the two most tractable variables, which achieved a progressive correspondence to guidelines in the early years of the registry), the expected percentage degree of compliance remained undefined. Indeed, there was a withdrawal from quantification in one area on the plausible basis of clinical complexity (e.g. haemodialysis blood pressure) and incomplete patho-physiological understanding. Such vagueness arguably undermined the credibility of the guideline advice. The relative incapacity to change somatic homeostatic variables was matched to uncertainty about the beneficial consequences of achieving clinical performance measures [20]. A range of success and failure was accepted. In the event, guideline compliance with metabolic variables in dialysis patients has settled over the past decade at something under two-thirds in point prevalent distributions. It appears that is typical of clinical compliance in Medicine generally [21]. However, the UKRR has been able to track continuously the change of Haemoglobin outcomes in response to a varying international specialty consensus, and the early increase in URR towards a stable performance, over 25 years (N.B. the UK had eschewed the measurement of haemodialysis dose by Urea Kinetic Modelling or formula in favour of URR) [22].

**Fig. 1:**
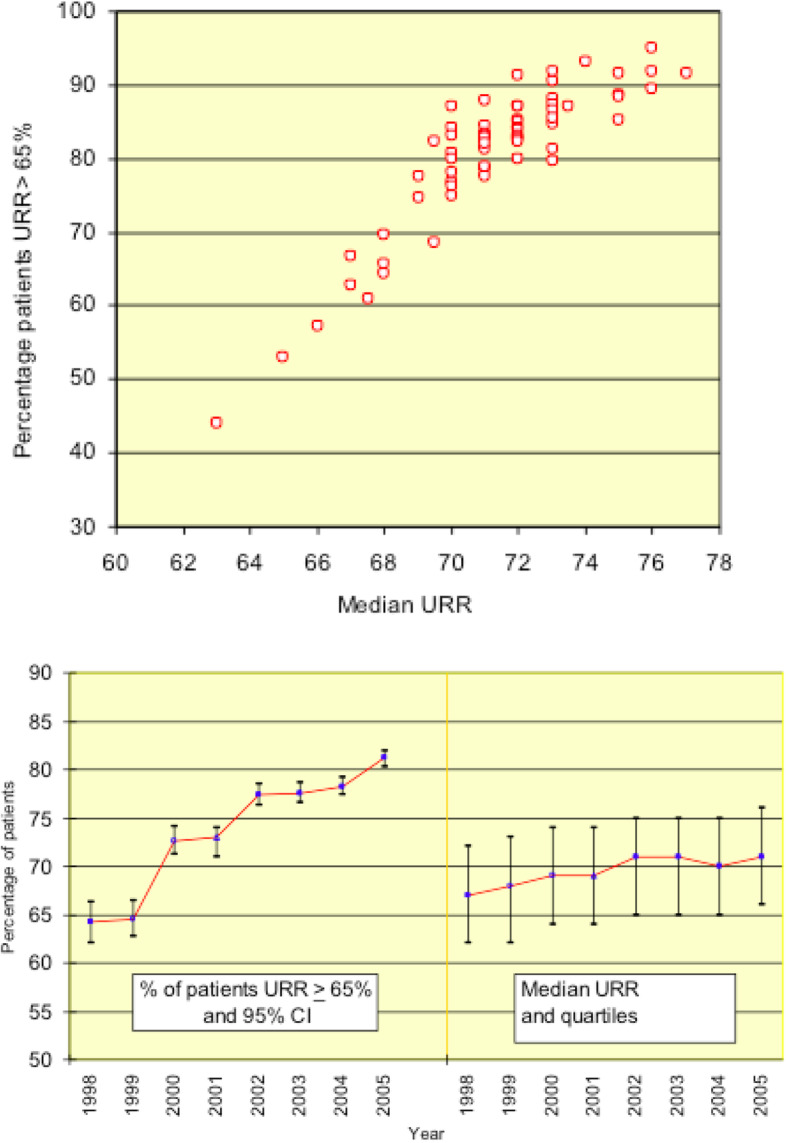
Haemodialysis data from reporting renal units in the Ninth UKRR Report. Urea Reduction Ratios % (URR) as a measure of treatment adequacy [Renal Association Standard > 65%]. Top panel Each Unit Population % > 65 Vs Unit Median Values: 2005 data. Lower panel Total population % > 65 and averaged medians: 1998–2005

The optimism that feedback of audit data would promote corrective action has been the subject of academic examination and is far from validated [23, 24]. In practice, the early RA default assumption, that closure of audit cycles would be attempted at unit level, left some discomfort in the UKRR [25]. Formally, it was not expected to contribute to sustaining the audit cycle, but annual UKRR user meetings and the formats of data presentation seemed relevant to that purpose. The UKRR Reports before 2011 (Report 14) attempted indirect guidance of clinical aspiration through Rose-Day graphics when there was wide dispersion of results (Fig. 1), although that contribution was, with exceptions, poorly understood by clinicians [2, 26, 27]. Registry experience did demonstrate the particular importance of unit Mean/Median values for achievable compliance with single guideline thresholds (as in Fig. 1) and Standard Deviation for compliance with a pre-defined, desirable range of values. More orthodox CQI-style software facilitations were offered in the 2010 Report, in similar passive support to the clinical periphery (Report 13, Ch 14) [2].

In an incipient climate of CQI the registry, as a presentational outlet of national renal reviews, was reinvigorated by an influx of data on haemodialysis vascular access (2005, Report 8, Ch 6) and infections (2008, Report 11, Ch 12) [2]. This was an enlargement of scope in the face of US evidence of access related co-morbidity, at a time when the limited potential of the laboratory components was becoming appreciated [28]. More recently, NHS agencies took up the novel categories of CKD (surveyed first in 2004, Report 7, Ch 3) and AKI [2]. National data collection initiatives were proposed, with the registry as the candidate repository for data to compile AKI incidence (2014, Report 17, Introduction) and the prevalence of stage 4/5 CKD (2016) [2]. This second invigoration, divorced from the unit-based IT infrastructure, depends on new linkages for data acquisition. The first fruit of this has been the 2020 UKRR Report on AKI laboratory warning scores in England (2017–18 data) [29]. When these pathways are mature, the UKRR should be able to move beyond the descriptive, to ground fully the epidemiologies of AKI and CKD (in terms of prevention and mitigation), just as it allowed previously the unpacking of concerns about listing for renal transplantation [1]. In the meantime, with the transplant data in hand, the stages of CKD have been introduced in the matrix of post-transplant results (Report 21, Ch 5, Table 5.8) [2]. National clinical leaders encouraged the efforts to focus on the renamed renal failure conditions and recruited NHS support.

The exchange of materials has been two-way, with the registry taking advantage of national Hospital Episode Statistics (HES) casemix data to compensate for the ‘stubbornly incomplete’ renal unit returns of patient comorbidity and mortality (Report 14, Ch 13) [2]. In general, the registry has been better placed to present an annual, if delayed, national position statement than fill-in the clinical specifics, like medication, which might explain it. The What has been, as ever, more accessible than the How or indeed the Why. Given the delayed, patchy, provision of hospital-wide electronic patient records in the NHS, it may be supposed that IT-gifted laboratory results are simply less demanding of unit data acquisition and entry than clinical details. Achieving any change to the basics of reporting has been slow at peripheral unit level. The scrupulous performance of units in Northern Ireland, for example, suggested that both size and culture may be relevant. The persisting patterns of unit returns, their accuracy and completeness, often seem to stem from the historical ad hoc development of UK renal clinical IT, the recapitulating Ontogeny reflecting Phylogeny [5].

The registry epidemiological databank has been exploited extensively and effectively in recent decades for academic Health Services Research (HSR), related especially to the social determinants of health and access to treatment [1]. That work has offered a continuing substrate to Kidney Research UK [1]. Renal replacement techniques have long been associated with extending the roles of nursing staff and close, long-term relationships with patients. The post-millennial social movement to promote patient involvement was addressed after 2006 by the free patient access to unit clinical databases proffered by the ‘Renal Patient View’ program, with the UKRR as a later mooring–point for that project (2011). Subsequently, a registry patient council was inaugurated (2014, Report 17, Foreword). Several registry-linked patient surveys are in hand, in a contemporary effort to characterise patient experience, needs and preferences. The universal replacement of ‘kidney’ for ‘renal’, and simplified reporting, have resulted from the egalitarian effort to interpret clinical issues to a general audience.

As another adjunct, those interested in ‘orphan’, rare, renal diseases have come together in the RaDaR initiative as a bolt-on to the UKRR platform (2011). The registry offered a locus and professional credential to these otherwise independent coalescences. By contrast, several modern issues have not yet attracted either advocacy, reporting mechanisms or study, including ‘frailty’, the context of the end of life and commercial dialysis outcomes. A study of the conservative management of end stage kidney disease is in hand.

Data reception, validation and presentation through an annual report were the staple and ballast of the registry project, with limited early efforts to communicate specific findings more conveniently (in registry ‘dips’ of selected information, for example) before a greater realisation of the internet [1]. However, the original professional template was transformed latterly by initiatives growing out of the non-governmental follow-up to the R-NSF, reported as ‘Kidney Health: Delivering Excellence’ (2013). These have taken advantage of modern informatics as a means of wider communication and exchange. A joint programme with NHS England, marketed as ‘Think Kidneys’, provides a virtual agency dealing with a pot-pourri of AKI, CKD and QI issues from cyberspace [30]. This now promotes particularly the patient-centricity of the NHS Long Term Plan (2019), as well as both longstanding and current renal issues. It remains to be seen what techniques the registry will deploy to support the unfolding national effort of multidisciplinary renal QI/CQI. The pitch and content of the annual UKRR reports has already been simplified (2018, Report 21) and further exploratory formats should be expected, in conjunction with the efforts at journal publication [2].

While Quality ‘is the only organising principle of renal care’ (2010) (Report 13, Foreword) renal CQI has a chequered history over two decades [2, 31, 32]. The learning curve is reflected in the report of the partially successful multi-agency research projects of the past few years, which also carries a virtual handbook of CQI [33]. Evaluation remains something of an Achilles Heel as the basis of a general introduction of change [34]. The ASSIST-CKD project is an encouraging example of a continuing partnership of CQI with EBM, reflecting the need to balance a predominant consensus with a traditional pluralism [33]. The necessary substrates to CQI methodology are not always apparent [14, 35]. Anyway, out of these dynamics has come a welcome, overdue, enhancement of the status and involvement of non-medical renal professionals in the CQI ‘collectives’, as well as a consolidation and modelling of patient involvement.

The democratisations have not been universal. The failure to establish coherent NHS IT, and the ultimate disregard for the IT component of the R-NSF, has prompted a specialty specific IT project (UKRDC 2013-), designed to pilot renal data acquisition with subsequent distribution to special interest groups including the UKRR (see Report 20, Introduction) [2]. Those who can, do. However, peripheral renal unit IT provision and infrastructure, the voluntary source of all the patient and unit data, remains unsupported (and unincentivised) from the NHS centre. It is those renal systems that have demonstrated the possibility, and possibilities, of everyday clinical data collection since the 1980s, of which the most recent are UKRR-based national RCTs [5]. An extension of that reach into digitally-acquired patient data will be a challenging stretch, if that is the future of the registry [36].

The shape of that future seems likely to be more dependent on outside influences than was the individual furrow ploughed by the early registry. There seemed enough to do in solving the communication and technical issues of the chosen modus operandus. In retrospect there was no liaison with the then British Computer Society (BCS), for example, or the other early UK registry, ICNARC (1995). There was modest early academic linkage, which became more extensive as the material for HSR and scholarly research in QI burgeoned [1]. Overseas registry activity in the specialty was acknowledged and compared in episodic international chapters of the Annual Reports (see Reports 2, 3, 6, 9–12), becoming less detailed and more epidemiological with time [2]. At the millennium, EDTA-R was reconstituted as ERA-EDTA-R to compile European RRT statistics, with less emphasis on collateral treatment issues than the original. If anything, the UKRR was the useful model, rather than the other way round [25, 37]. The updated Primary Renal Disease codes of the ERA-EDTA (2009) were adopted by UKRR (2015, Report 18). The Lombardy Registry experience in northern Italy was pursued in a parallel, long-term, time frame, albeit on the somewhat smaller scale of 44 renal units (1982). It also has brought together clinical data and treatment epidemiology [38]. That comparison emphasises the significance of the incidental UK scale of the registry task, 71 adult units, in offering ready communication and manageability together with statistical power. The much larger USRDS functions with regional networks for the epidemiology of RRT and (after 2003) CKD, but then data collection is based on quite different principles, which makes useful comparison difficult [39]. The US nationwide ESRD Clinical Performance Measures Project has concentrated since 1994 on dialysis adequacy, Serum Albumin and dialysis access and has not been beguiled by the availability of other routine laboratory data. The practical relevance of scale of operation is apparent in other sustained systems, like the ANZDATA registry (1977, reporting regularly after 1997) [40]. While the DOPPS programme was scrutinised in early days, its sampling approach to practice patterns was open-ended, compared to the UKRR remit [41]. The idiosyncratic capacities of the UKRR are based on an NHS that has provided, and continues to provide, a unique context for complete data collection and joint interests.

By contrast, internal organisational elements of the UKRR have tended to copy external social and business styles. The early exploratory phase was catalysed by dedicated appointments and was dependent on the commitment of a few clinical enthusiasts, working voluntarily from NHS posts. The necessary transition to a settled, more routine, contribution after 2007 prompted yet closer integration with RA organisation, formal commitments and the appointment of a Chief Executive (2011) [1]. Subsequent appointments have reflected the proliferation of research, programmes and collaborations.

## Conclusion

In hindsight, the UKRR has been moved irregularly with the times to reflect not only the professional re-evaluations of international and UK renal medicine but also NHS preoccupations. The broader informatics net (a modern web portal has been established (2019)) is suited to the post-millennial shift in Nephrology, from an emphasis on palliation towards prevention. As a ‘register’ it would have been a less comfortable chameleon, but the flexible registry construct has been shaped ultimately into a platform responsive to both RA and NHS purposes.

The hybrid UKRR agency is arguably one of at least three major compromises that have emerged over the several decades to permit constructive momentum in the specialty and the NHS. The changes can be seen as acceptable responses to the many demands made on the complex adaptive system (CAS) of a national specialty [42]. The R-NSF was based on the first compromise, the introduction of a renal ‘Tsar’. It signalled a quite new authority in the form of a plausibly bi-partisan National Clinical Director of Kidney Care. The R-NSF served in retrospect as a post-millennial bridge to contemporary specialty means and ends. In a gradual second compromise, the deep professional instinct for patient benefit expressed as quality improvement has for the most part absorbed its modern methodologies. Recent compound semantics (‘evidence-based improvement’, ‘evidence-informed implementation framework’) declare an unselfconscious melding of the disciplines of EBM, Audit-feedback and CQI in the clinical periphery [43]. The third development is the refashioning of the current registry as a multi-faceted agency. Its status as a formal entity, with stable infrastructure, has offered a useful base to a variety of special interests. The modern preoccupation with the detail of patient experience has prompted registry exploitation of social media, the Internet and patient surveys in conjunction with non-medical agencies. These various liaisons have moved registry interests well beyond the initial medical remit. It remains to be seen just what place it will come to take in what has been called a ‘maze’ of NHS indicators [44].

The three hybridisations have accommodated the post-millennial optimism of a specialty characterised otherwise by much repetitive labour. It is easy to see why the authors of Forewords to the modern Annual Reports convey unstinting admiration, as an expression of the reconciliation of professional and bureaucratic vital interests within the NHS [1, 2].

Of the specific features, the profiles of access to treatment and provision, informed by the expanded epidemiological elements of the registry, are being established by specialty HSR [1]. Arguably, kidney healthcare ‘Delivery’, dependent on staffing and physical resources, has yet to be fully incorporated as one of the many putative dimensions of the renal Quality matrix of the NHS [31]. Of course, that is just where the 1995 registry came in [1]. Nevertheless, it remains a well-placed vehicle for specialty development, with the KQuIP initiative as an example of the progressive networking and inclusivity offered by modern informatics to the organisation of post-pandemic national healthcare [36, 45].

## Data Availability

Not applicable.

## Abbreviations

AKI: Acute Kidney Injury
ANZDATA: Australia & New Zealand Dialysis & Transplant Registry
BCS: British Computer Society
CAS: Complex Adaptive System
Ch: Chapter
CKD: Chronic Kidney Disease
CQI: Continuous Quality Improvement
DOPPS: Dialysis Outcomes and Practice Patterns Study
EBM: Evidence Based Medicine
EDTA-R: European Dialysis and Transplant Registry
ERA-EDTA-R: Registry of the European Renal Association- European Dialysis and Transplant Association
ESRD: End Stage Renal Disease
HES: Hospital Episode Statistics
HSR: Health Services Research
ICNARC: Intensive Care National Audit & Research Centre
IT: Information Technology
KQuIP: Kidney Quality Partnership
NHS: National Health Service
QI: Quality Improvement
RA: Renal Association
RCP: Royal College of Physicians (London)
RCT: Randomised Controlled Trial
R-NSF: Renal National Service Framework
RRT: Renal Replacement Therapy
UK: United Kingdom
UKRDC: United Kingdom Renal Data Collaboration
UKRR: United Kingdom Renal Registry
URR: Urea Reduction Ratio
US: United States of America
USRDS: United States Renal Data System
